# Technology Acceptance and Usability of a Virtual Reality Intervention for Military Members and Veterans With Posttraumatic Stress Disorder: Mixed Methods Unified Theory of Acceptance and Use of Technology Study

**DOI:** 10.2196/33681

**Published:** 2022-04-21

**Authors:** Chelsea Jones, Antonio Miguel Cruz, Lorraine Smith-MacDonald, Matthew R G Brown, Eric Vermetten, Suzette Brémault-Phillips

**Affiliations:** 1 Heroes in Mind, Advocacy and Research Consortum Faculty of Rehabilitation Medicine University of Alberta Edmonton, AB Canada; 2 Leiden University Medical Centre Leiden Netherlands; 3 Edmonton Occupational Stress Injury Clinic Alberta Health Services Edmonton, AB Canada; 4 Glenrose Rehabilitation Hospital Research Innovation and Technology Glenrose Rehabilitation Hospital Alberta Health Services Edmonton, AB Canada; 5 Department of Occupational Therapy Faculty of Rehabilitation Medicine University of Alberta Edmonton, AB Canada

**Keywords:** PTSD, UTAUT, technology acceptance model, trauma, mental health, therapy, rehabilitation, digital health, psychotherapy, military, veteran, psychotherapy, 3MDR, technology acceptability, technology acceptance, Canadian Armed Forces, virtual reality

## Abstract

**Background:**

Military members and veterans exhibit higher rates of injuries and illnesses such as posttraumatic stress disorder (PTSD) because of their increased exposure to combat and other traumatic scenarios. Novel treatments for PTSD are beginning to emerge and increasingly leverage advances in gaming and other technologies, such as virtual reality. Without assessing the degree of technology acceptance and perception of usability to the end users, including the military members, veterans, and their attending therapists and staff, it is difficult to determine whether a technology-based treatment will be used successfully in wider clinical practice. The Unified Theory of Acceptance and Use of Technology model is commonly used to address the technology acceptance and usability of applications in 5 domains.

**Objective:**

Using the Unified Theory of Acceptance and Use of Technology model, the purpose of this study was to determine the technology acceptance and usability of multimodal motion-assisted memory desensitization and reconsolidation (3MDR) on a virtual reality system in the primary user group (military members and veterans with treatment-resistant PTSD, 3MDR therapists, and virtual reality environment operators).

**Methods:**

This mixed methods embedded pilot study included military members (n=3) and veterans (n=8) with a diagnosis of combat-related PTSD, as well as their therapists (n=13) and operators (n=5) who completed pre-post questionnaires before and on completion of 6 weekly sessions of 3MDR. A partial least squares structural equation model was used to analyze the questionnaire results. Qualitative data from the interviews were assessed using thematic analysis.

**Results:**

Effort expectancy, which was the most notable predictor of behavioral intention, increased after a course of 3MDR with the virtual reality system, whereas all other constructs demonstrated no significant change. Participants’ expectations of the technology were met, as demonstrated by the nonsignificant differences in the pre-post scores. The key qualitative themes included feasibility and function, technical support, and tailored immersion.

**Conclusions:**

3MDR via a virtual reality environment appears to be a feasible, usable, and accepted technology for delivering 3MDR to military members and veterans who experience PTSD and 3MDR therapists and operators who facilitate their treatment.

## Introduction

### Background

Military services commonly involve engagement in high-risk activities, whether during physical training, daily trade-related tasks, overseas deployment or in response to natural disasters. Such activities place military members, individually and collectively, at a heightened risk of physical and psychosocial injury. Canadian Armed Forces (CAF) military members and veterans exhibit higher rates of injuries and illnesses, such as posttraumatic stress disorder (PTSD), major depressive disorder, generalized anxiety disorder, substance abuse, sleep disorders, and mild traumatic brain injury compared to their civilian counterparts [1,2]. These conditions can have far-reaching implications such as occupational, social or familial, and psychological impairment and can affect activities of daily living. Numerous studies conducted in Canada, the United States, and the United Kingdom have demonstrated a high prevalence of PTSD specific to deployments during conflicts in the Middle East from 2001 to 2013 [1-5]. The rate of probable PTSD among UK military personnel has been reported to be 6.2% [3] and, among veterans who were deployed in combat roles, 17.1% [4]. The rate of PTSD among Canadian veterans is estimated to be 16% [5]. Owing to the prevalence of these mental health conditions among military personnel and veterans, evidence-based interventions and treatments are needed to assist in recovery and rehabilitation. As our understanding of trauma evolves, novel interventions for PTSD in this population are needed. In particular, the use of technology as a facilitator of treatment may introduce avenues of recovery that were not previously possible.

### Multimodal Motion-Assisted Memory Desensitization and Reconsolidation

Multimodal motion-assisted memory desensitization and reconsolidation (3MDR) is an innovative, technology-assisted, exposure-based trauma therapy that holds promise for treating combat-related PTSD (crPTSD). 3MDR is a structured, personalized, exposure-based, virtual reality (VR)–supported intervention developed in the Netherlands and used with military members and veterans with PTSD in the Netherlands, the United Kingdom, the United States, Israel, and Canada [6]. 3MDR is an emerging VR-assisted therapy delivered in an immersive VR environment (VRE) such as the Motek Gait Realtime Analysis Interactive Lab or Motek Computer Assisted Rehabilitation Environment (CAREN). The most commonly used VRE for 3MDR has been CAREN, which is a room-sized, 3D VRE with a central treadmill surrounded by 240° floor-to-ceiling motion-capture screens.

The 3MDR intervention comprises 10 sessions, including selecting images and music, trauma processing, and reconsolidation, and six 90-minute therapy sessions in the VRE, including a 30-minute debrief [7]. The 3MDR sessions include a *preplatform session* (session 1), during which the participant selects and orders images and music. Symbolic representations in the form of images (ie, photographs and sketches) related to their traumatic experiences are selected and ordered from least to most distressing. Music that reminds the participant of the traumatic event or events and facilitates the emotional memory network is also identified, which supports a return to the present. Sessions 2 to 7 are *platform sessions* that involve 3 phases. In the *preplatform phase* of the session, the therapist and participant confirm the order of the images and music for the session. During the *platform phase*, the participant dons a safety harness and is accompanied by a 3MDR therapist while walking continuously on a treadmill at a self-selected pace. The participant first warms up by walking on the treadmill while listening to self-selected music connecting them to traumatic experiences and then, during each of seven 3- to 5-minute cycles, walks down a 3D hallway on the screen toward a self-selected trauma-related image. The participant describes the image, physical sensations, and feelings, followed by communicating descriptive words and phrases with the help of a therapist. These words and phrases are projected in front of the image and then read aloud by the participant. For a duration of 30 seconds, the participant then reads aloud numbers as they appear on a ball oscillating horizontally in the foreground of the image and words. The participant cools down after the seventh cycle by walking while listening to self-selected music, which facilitates reconnection to the present. Each session is concluded with a *postplatform phase*, which includes discussion, reconsolidation, and a mental wellness check or self-care plan. *Postplatform session*s 8 to 10 focus on reconsolidation and contribute to the meaning making of the acquired gains [7]. In-depth descriptions of 3MDR have been published elsewhere [6,7,8].

Initial randomized controlled trials with 3MDR participants have shown a reduction in PTSD symptoms, which was maintained over time [9,10]. Although these results indicate that 3MDR may be a promising new therapeutic treatment for PTSD, key areas of exploration are required before 3MDR can be implemented as a frontline trauma modality. One such area of needed exploration is the technology acceptance and usability of 3MDR from the perspective of the end users, including CAF military members and veterans with crPTSD and the therapists and operators delivering 3MDR. Questions on feasibility must be addressed before in-context clinical investigations regarding specificity, reliability, validity, and sensitivity can take place. Without addressing acceptance and usability, technological innovations may not be adopted or implementations sustained. For 3MDR specifically, the combination of a military context and the intervention potentially affects multiple user levels; careful exploration to determine whether the technological components of 3MDR are acceptable and beneficial is warranted.

### Technology Acceptance and Usability in Contexts

Technology offers health care professionals a variety of benefits, including improving the efficacy, efficiency, safety, and cost-effectiveness of assessments, interventions, data collection, data analysis, reporting, record keeping, and communication. The acceptance of such technologies by health care professionals is an important topic for health care professionals and researchers [11,12]. Without technology acceptance and usability for the user, technological assessments, interventions, and other aspects that would assist with evolving health care needs may not be adopted into clinical practice despite their potential effectiveness. Therefore, evaluation of the acceptance and usability of emerging technology is integral to advancing best practices in health care [12].

The use of digital and mobile health innovations is becoming widespread in military and veteran populations [13, 14-17]. This has been amplified by the recent COVID-19 pandemic, when web-based health solutions have become increasingly common in all health care practices, including military environments [14-17]. It is essential to, directly and indirectly, assess the technology acceptance of different user groups, including military personnel, within their context using a framework or model to ascertain this factor, which contributes to the feasibility of implementing technological innovations.

### Purpose

The purpose of this mixed methods pilot study is to use the Unified Theory of Acceptance and Use of Technology (UTAUT) model to determine the technology acceptance and usability of 3MDR within a VRE in the end user groups of (1) military members and veterans with crPTSD, (2) 3MDR therapists, and (3) VRE operators. On the basis of previous research in this area, it is hypothesized that performance expectancy (PE) and facilitating conditions (FC) will be the most influential variables on behavioral intentions (BI) and use, respectively. In addition, it is hypothesized that social influence (SI) will have the least influence on BI.

## Methods

### Study Design

This study used a mixed methods, embedded study design with a pre-post quasi-experimental approach. A quantitative approach using partial least squares structural equation modeling (PLS-SEM) and other nonparametric statistics was the primary method of data collection; a qualitative thematic analysis was secondary to this. This study was embedded in a larger study that used a mixed methods staggered entry clinical trial to test the efficacy, effectiveness, and safety of 3MDR [8].

### The UTAUT Model

**Figure 1:**
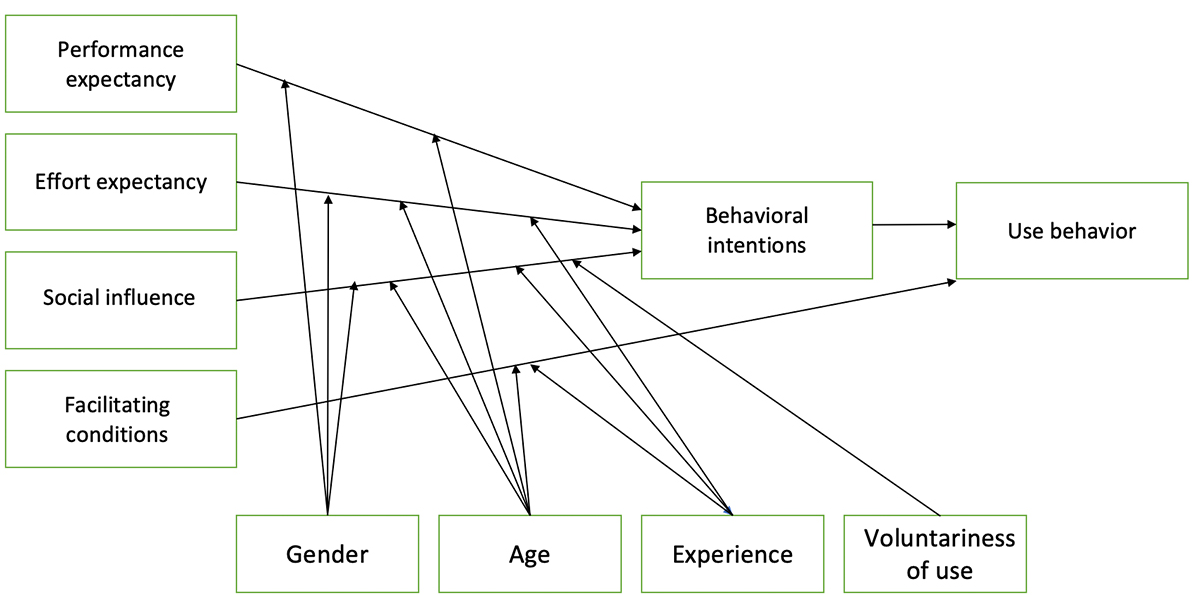
The Unified Theory of Acceptance and Use of Technology Model [18].

The UTAUT model was developed based on previous theories and models for the acceptance and adoption of technologies and consumer products that address the perceived technology acceptance of a user group with the goal of predicting use behavior (Figure 1) [18]. UTAUT has been demonstrated to explain as much as 70% of the variance in the intention to use technology compared with its technology acceptance model predecessors [18].

The UTAUT model addresses the perceived expectations of technological acceptance of new technology in five constructs: PE, effort expectancy (EE), and SI (direct determinants of BI), as well FC and BI, which have a direct impact on use behavior [18]. This model was developed from the point of view of the implementation of new technologies in practice within specific organizations rather than the technology for mass consumer consumption [18-20]. The UTAUT is a model that is commonly tested using PLS-SEM and is an example of a reflexive partial least square (PLS) path model [18]. The exogenous latent variables (PE, EE, and SI) have an effect on the endogenous latent variable (BI), which in turn affects the construct of use [18]. In addition, FC can also have a direct effect on use [18]. Moderator variables, which include age, gender, experience, and voluntariness of use, also affect the interaction between indicators and constructs [18,19].

BI is defined as the intention to use technology, and use is defined as the actual use [18]. BI predicts whether the technology in question will be adopted by the user in reality. The three direct determinants of BI to use technology are PE, EE, and SI. PE is defined as the degree to which an individual believes that using the system will help a person attain gains in task performance [18]. The EE construct is defined as the degree of ease associated with the use of the system, and SI is the degree to which an individual perceives the importance of others believing that they should use the new system [18]. FC is defined as the degree to which an individual believes that an organizational and technical infrastructure exists to support the use of the system [18]. FC, PE, and EE are considered beliefs or the information a person has about an object, and SI is considered the subjective norm [18]. The UTAUT has well-established construct and content validity.

The UTAUT has been used in recent years as a model and framework for addressing technology use and acceptance in health care [11,12,21]. To date, most research on health technology using the UTAUT has involved the exploration of computerized medical records where the primary intended user, or end user, is a health care professional [11,12]. Studies that focus on the patient as the end user are beginning to emerge in the literature for specific demographics, such as older adults and youth, as well as specific populations with specific diagnostic categories such as cardiovascular disease, mental health, and diabetes. These studies evaluated the technology acceptance and usability of a multitude of digital and mobile health technologies, including health apps, wearable measurement technology, augmented reality, and web-based access to medical records. Hypotheses regarding the effect of the latent variables on BI and use have been formed regarding health care professionals as end users [11]. Studies focusing on the patient as the end user have demonstrated variable results, making the formation of a directional hypothesis challenging. In addition, studies examining technology acceptance models among military personnel are scarce [22,23].

### Sample Eligibility and Size

The target study sample size was set at a minimum of 40 military members and veterans to account for a 20% dropout rate and allow for power at 32 participants. With 4 latent variables, for 80% significance at a 5% significance level, the sample size required for this study was 24 (*R*^2^=0.50) [24].

### Recruitment and Sampling

Recruitment of regular and reserve CAF military members and veterans was conducted by word of mouth among potential participants and their mental health providers as convenience and snowball sampling. Service providers supporting CAF military members and veterans, after being informed of the study via word of mouth and institutional email, informed patients who met the study inclusion and exclusion criteria. Potential participants who showed interest in participation were provided with a *Permission to Share Contact Information with the Research Team* form by their service provider. The completed forms were then forwarded to the research team. The researchers then contacted the potential participants via phone or email with a request for them to meet with the research team to learn more about the study and be evaluated to confirm eligibility to participate. Voluntary verbal and written informed consent were obtained from all CAF military members and veterans participating in the study.

Recruitment of the 3MDR therapists and operators was initiated via email circulated by key stakeholders associated with the 3MDR studies at 7 sites within Canada, the Netherlands, the United Kingdom, and the United States. 3MDR therapists and operators interested in participating in the study were instructed to email the research team to indicate consent to be contacted. Participants who met the inclusion criteria were forwarded a web-based consent form via a secure server (REDCap [Research Electronic Data Capture]) or hard copy, and an interview time was scheduled. Potential participants were informed that engagement in the study was voluntary.

### Ethics Approval

This study received approval from University of Alberta Research Ethics Board (Pro00084466) and CAF Surgeon General Research Program (E2019-02-250-003-0003).

### Inclusion and Exclusion Criteria

The 3MDR study participants included regular and reserved CAF military members and veterans aged 18 to 60 years under the care of a mental health clinician or service provider working at or associated with a Canadian Forces Base, an Operational Stress Injury Clinic, or Veterans Affairs Canada. All participants met the Diagnostic and Statistical Manual–Fifth Edition [25] criteria for PTSD diagnosis and had a score of ≥30 on the Clinician-Administered PTSD Scale for the Diagnostic and Statistical Manual of Mental Disorders–Fifth Edition Worst Month version. Participants were required to be stable on their current psychotropic medication for at least 4 weeks before entering the study. Individuals with comorbidities were included if they satisfied other inclusion and exclusion criteria. The participants were English speaking and able to provide informed written consent. The detailed 3MDR protocol has been previously published [8].

The 3MDR therapists and operators included in this study were English-speaking current or previous 3MDR therapists and operators who were trained by the developer of 3MDR. Participants must have completed a full course of 3MDR delivery with at least one patient (ie, had completed six 3MDR platform sessions using a VRE).

### Measurements and Instruments

A demographic questionnaire was provided via email to participants through the REDCap server or in hard copy form. Variables collected from patient participants included age, sex, marital status, employment status, military status, enrollment era, rank, element, and years of service. For the 3MDR therapists and operators, the collected variables included the participants’ sex, profession, role in delivering 3MDR, years using 3MDR, location, VRE used, and level of education.

Two UTAUT questionnaires specific to the end users were developed specifically for this study. Version 1 (time point 0 [T0]) included questions in the future tense, whereas version 2 (time point 1 [T1]) included the same questions but was modified to reflect the past tense. The 12 questions’ outcome measures were based on a Likert scale, with a score of 1 to 7 assigned to each question, with 1 being *strongly disagree* and 7 being *strongly agree*. A Likert scale with 7 points was used as the original UTAUT questionnaire by Venkatesh et al [18] used a 7-point scale. The maximum and minimum scores were 105 and 15, respectively. The 15 included questions addressed the five different constructs of the UTAUT (n=3, 20% PE; n=3, 20% EE; n=3, 20% SI; n=3, 20% FC; and n=3, 20% BI) that influence the use of technological innovations. Gender and age demographic information were also collected via the UTAUT questionnaire as they are modifier variables within the UTAUT model. The UTAUT questionnaire was provided only to those participants in the Canadian arm of a larger study who used the CAREN as the VRE.

### Data Collection

The UTAUT questionnaires were completed by patients, therapists, and operators before and after 6 sessions of the 3MDR. The questionnaires were administered by a member of the research team before the qualitative semistructured interviews. Version 1 of the UTAUT questionnaire was presented before its first introduction to the CAREN and 3MDR. This version was future tense oriented and intended to measure expectations of the technology. After completing this questionnaire, the participants engaged in 3MDR for 6 sessions over approximately 6 weeks before completing the version 2 UTAUT questionnaire. This version was written in the past tense, intending to measure the actual intention to use technology once the participants had some experience with it.

A semistructured interview guide was developed to collect qualitative data. The research team conducted individual 40- to 60-minute semistructured interviews either in person or via telephone or a secure Zoom videoconferencing platform with the 3MDR patients, therapists, and operators. All interviews were recorded and subsequently transcribed by the research team.

### Data Analysis

The research team conducted both quantitative and qualitative analyses. Quantitative analysis was based on the UTAUT, which uses a reflexive path model and PLS-SEM. The expectations from T0 and actual experience from T1 were statistically analyzed using PLS-SEM with a within-sample path model. Structural equation modeling (SEM) is considered a second-generation technique of multivariate analysis that allows researchers to incorporate unobservable variables measured indirectly by indicator variables [26]. PLS-SEM is variance based, as it accounts for the total variance and uses this to estimate the parameters [27]. In this method of analysis, the algorithm computes partial regression relationships in the measurement and structural models using ordinary least squares regression [26,27]. In an exploratory study such as this, data analysis is concerned with testing a theoretical framework from a prediction perspective, making PLS-SEM an ideal method for analysis [27].

The path model must be analyzed through measurement and structural model assessments [26,27]. Reflexive measurement models were evaluated based on internal consistency (Cronbach α), convergent validity (average variance extracted [AVE]), and discriminant validity (cross-loading analysis, Fornell-Lacker Criterion Analysis, and heterotrait-monotrait ratio) [26]. Evaluation of the structural model included an analysis of collinearity, significance, coefficients of determination (*R*^2^), size and significance of the path coefficients, effect size (*f*^2^), and predictive relevance (*q*^2^). Goodness of fit was not assessed as this was an exploratory PLS path model with both reflexive (measurement model) and formative (structural model) components, rendering current model fit measurements unnecessary and inappropriate [28].

As PLS-SEM does not assume that data are normally distributed—it relies on a nonparametric bootstrap procedure to test the significance of the estimated path coefficients in PLS-SEM. With bootstrapping, subsamples are created with randomly drawn observations from the original set of data (with replacement) and then used to estimate the PLS path model [28].

SmartPLS [29] was used for the PLS analysis. The maximum iterations were set at 300 with +1 as the initial value for all outer loadings, and the path weighting scheme and the stop criterion at 1×10^7^. Basic bias-corrected bootstrapping was used with 1000 samples at a significance level of *P*<.05. SPSS (2017) [30] was used for the analysis of descriptive statistics (mean and SD), and frequency counts, the Harman single-factor test, and a Wilcoxon signed-rank test was used to detect before and after changes in scores [31,32]. Webpower [33] was used to verify the nonnormality of the data before the analysis.

Qualitative interview data were subjected to thematic analysis (inductive and deductive) to identify, analyze, and report patterns (themes) in rich detail and allow the researcher to interpret various aspects of the topic [34]. Although inductive analysis allowed for themes to emerge from the data, deductive analysis was guided by the research questions regarding the perceived technology acceptance and usability of 3MDR among end users, including the perceived strengths, weaknesses, and recommendations for future use. Following a review of the data and completion of the secondary level of analysis, the themes were narratively summarized with the aim of organizing, describing, exploring, and interpreting the data. Key quotations were selected to substantiate these findings. To ensure the validity, reliability (dependability), and conformability of the analysis, researcher bias was clarified and bracketed, and an external audit of the analysis was conducted by other members of the research team [35-37]. The main theme with 3 subthemes emerged through thematic analysis of the data, which was relevant to the question of technology acceptability.

A concurrent parallel approach following a data transformation model was used in the data analysis process to convert data to compare quantitative statistical results with qualitative findings [38].

## Results

### Demographics

A total of 29 end users of 3MDR participated in this study. The demographic information of the military (3/29, 10%) and veteran (8/29, 28%) sample is displayed in Table 1 and of the 3MDR therapists (13/29, 45%) and operators (5/27, 17%) in Table 2. Of the total sample, only some military members and veterans (9/11, 82%), 3MDR therapists (4/13, 31%), and 3MDR operators (2/5, 40%) had the ability to fill out the pre- and post-UTAUT questionnaires. All participants (N=29) participated in the qualitative interviews. The sample was largely composed of men (19/29, 66%), which prevented the use of gender as a moderator variable in the UTAUT research model. In addition, the age of participants (young or middle-aged) did not demonstrate an effect in the research model and was, therefore, removed from the final PLS model.

**Table 1:** Sample demographic information of the military and veteran sample (N=11).

Characteristics			Participants, n (%)
**Sex**			
	Female	1 (9)	
	Male	10 (91)	
**Age (years)**			
	30-39	2 (18)	
	40-49	6 (55)	
	50-59	3 (27)	
**Marital status**			
	Common law	2 (18)	
	Divorced	1 (9)	
	Married	5 (45)	
	Separated	1 (9)	
	Single	2 (18)	
**Employment status**			
	Employed	6 (55)	
	Unemployed	5 (45)	
**Military employment status**			
	Active military member	3 (27)	
	Veteran	8 (73)	
**Military enrollment era**			
	1976-1990	2 (18)	
	1991-2000	8 (73)	
	2001-2015	1 (9)	
**Rank**			
	Junior NCM^a^	6 (55)	
	Senior NCM	4 (36)	
	Unknown	1 (9)	
**Element**			
	Air	2 (18)	
	Land	9 (82)	
	Sea	0 (0)	
**Duration of military service (years)**			
	5-10	2 (18)	
	11-15	1 (9)	
	≥20	8 (73)	

^a^NCM: noncommissioned member.

**Table 2:** Sample demographics of 3MDR^a^ therapists and operators (N=18).

Characteristics		Participants, n (%)
**Gender**		
	Man	9 (50)
	Woman	9 (50)
**Location**		
	Canada	7 (41)
	The Netherlands	6 (35)
	The United Kingdom	3 (17)
	United States	2 (11)
**Profession**		
	Occupational therapist	1 (6)
	Clinical psychologist	6 (33)
	Nursing	1 (6)
	Mental health therapist	1 (6)
	Mental health chaplain	2 (11)
	Researcher	8 (44)
	Technician	5 (28)
**Military experience**		
	No	16 (89)
	Yes	2 (11)
**3MDR role**		
	Therapist	13 (72)
	Operator	5 (28)
**Experience with 3MDR (years)**		
	<1	5 (28)
	1-3	9 (50)
	3-5	3 (17)
**3MDR system**		
	CAREN^b^	12 (67)
	GRAIL^c^	3 (17)
	CAREN Light	2 (11)

^a^3MDR: multimodal motion-assisted memory desensitization and reconsolidation.

^b^CAREN: Computer Assisted Rehabilitation Environment.

^c^GRAIL: Gait Realtime Analysis Interactive Lab.

### UTAUT Analysis

The psychometric properties of the raw data of the survey items used to measure the latent variables are shown in Table 3. The difference between the means of the pre-post summative scores is a 0.82% increase. When mean pre-post total scores indicate <5% difference in change, it indicates that the expectations of the participants regarding technological innovation were met within the constructs tested [19]. Using a Mann-Whitney *U* test, no significant difference was found between the therapists and operators and participants (*P*=.55).

The results of the measurement model evaluation, including the factor analysis, internal consistency (Cronbach α), convergent validity (AVE), and composite reliability, are shown in Table 4 The factor indicators, known as the outer loadings or reflexive indicator loadings, should be ≥0.5, demonstrating that the indicator variable is a good measurement of the latent variable [26]. Only one outer loading for SI was below this threshold, indicating good indicator reliability (Table 4). All latent variables, with the exception of SI, demonstrated values >0.70 for both Cronbach α and AVE, which would indicate good validity and reliability of the latent variables [26,38]. Composite reliability is displayed in Table 4 for all values, with the exception of SI ≥0.7, which is acceptable.

To evaluate discriminant validity, cross-loading, Fornell-Larcker Criterion, and heterotrait-monotrait ratio (Table 5) were used. These measures demonstrated good discriminant reliability for all the latent variables. FC demonstrated the highest correlation with BI based on this analysis. Potential common method bias was assessed with the Harman single-factor test, yielding cumulative and variance loadings <50%.

**Figure 2:**
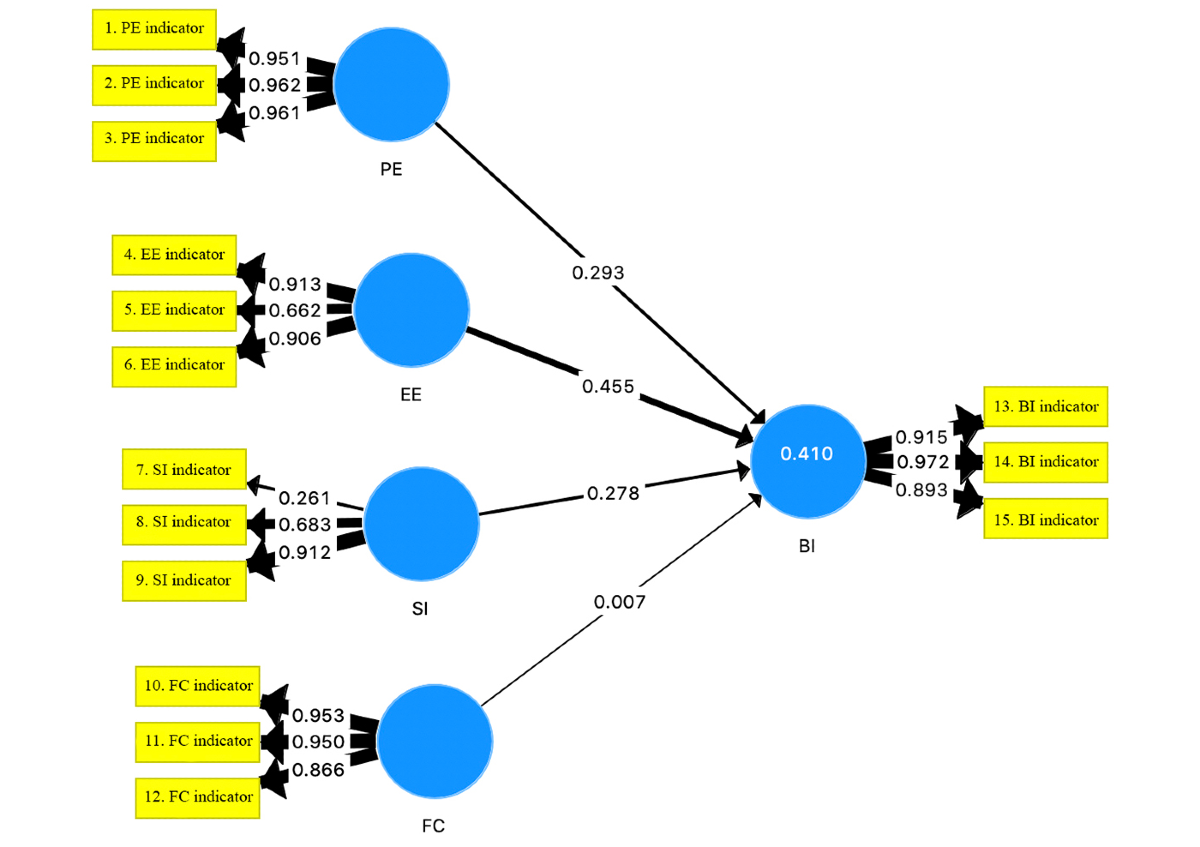
Partial least square path model; path analysis model of Unified Theory of Acceptance and Use of Technology predicting BI. *R*^2^=0.410. BI: behavioral intentions; EE: effort expectancy; FC: facilitating conditions; PE: performance expectancy; SI: social influence.

The measure of lateral collinearity of the structural model demonstrated inner variance inflation factor values <5 for 10 (66.67%) latent variables, with the exception of indicator variables number 1 (5.649), 2 (9.215), and 3 (5.410) for PE, 11 (5.584) for FC, and 14 (7.392) for BI. The coefficient of determination (*R*^2^) measures the proportion of variance in a latent endogenous variable that is explained by other exogenous variables, expressed as a percentage. The explained variance (*R*^2^) of the structural model was 0.410, demonstrating moderate predictive accuracy [26,38]. The effect sizes (*f*^2^) for each latent variable are presented in Table 6. On the basis of this analysis of the structural model, EE had the largest path coefficient and effect size, indicating that it was the strongest predictor of BI, although this was not significant (*P*=.40; Table 5 and Figure 2). The predictive relevance (*q*^2^) was >0 (0.026). None of the latent variables were statistically significant (*P*=.05).

A multigroup analysis with the PLS path model attempted to compare pre-post scores; however, this was not possible because of sample size restrictions. Instead, a Wilcoxon signed-ranks test was used to determine if there were any statistically significant changes in scores from before the technology was used (before to T0) to after the occurrence of the 3MDR course (after to T1). This showed a significant pre-post increase in the EE score only (*Z*=65; *P*=.004), with pre-post scores for all other variables yielding a nonsignificant change (Table 7). This demonstrates that the participants felt that the perceived ease of use of the technology was likely to increase after using 3MDR in the VRE, whereas the scores regarding the other latent variables remained largely unchanged from before to after.

**Table 3:** Psychometric properties of indicators used to measure latent variables.

Exogenous latent variables (indicators)		Values, mean^a^ (SD^b^)	Values, median^c^
**Performance expectancy (3 indicators)**			
	Using the CAREN^d^ system improved my medical condition (patient) Using the CAREN improved the medical condition of my patient (therapist and operator)	5.714 (1.082)	6
	Using the CAREN system had a positive effect on my medical condition (patient) Using the CAREN system had a positive effect on the medical condition of my patient (therapist and operator)	5.643 (0.961)	6
	The CAREN system improved my quality of life (patient) The CAREN system had improved the quality of life of my patient (therapist and operator)	5.357 (1.060)	6
**Effort expectancy (3 indicators)**			
	Interacting with the CAREN system was easy for me (patient, therapist, and operator)	6.429 (0.632)	6
	I believe my interaction with the system was clear and understandable (patient, therapist, and operator)	6.500 (0.516)	7
	I found the system easy to use (patient, therapist, and operator)	6.429 (0.516)	6
**Social influence (3 indicators)**			
	People who are important to me think that I should be involved in using the CAREN system (patient, therapist, and operator)	5.214 (1.496)	6
	I would use the CAREN system because my colleagues will use it too to improve their medical condition (patient) I used the CAREN system because my colleagues used it too to improve the medical condition of my patient (therapist and operator)	3.714 (1.944)	6
	In general, my organization has supported my involvement in this initiative (patient, therapist, and operator)	6.286 (1.290)	6
**Facilitating conditions (3 indicators)**			
	I believe guidance was available to me during my interaction with the CAREN system (patient, therapist, and operator)	6.571 (0.507)	6
	I believe specialized instruction concerning the interaction with the CAREN system was available to me (patient, therapist, and operator)	6.500 (0.640)	6
	A specific person (or group) was available for assistance with CAREN system difficulties (patient, therapist, and operator)	6.500 (0.834)	6
**Behavioral intentions (3 indicators)**			
	I am willing to use the CAREN system in the next weeks (patient, therapist, and operator)	6.571 (0.632)	6
	I plan I would use the CAREN system if I am willing to do so (patient, therapist, and operator)	6.071 (1.246)	6
	I predict I will use the CAREN system in the future (patient, therapist, and operator)	5.857 (1.438)	6

^a^Raw mean scores of items within the scale, where each item is measured on a 7-point Likert scale; 1=strongly disagree, and 7=strongly agree. The higher the indicator score, the more agreement with the statement.

^b^SD of raw scores.

^c^Median scores of each question.

^d^CAREN: Computer Assisted Rehabilitation Environment.

**Table 4:** Results of the validity and reliability evaluation of the measurement model.

Latent variables, indicator variables, and outer loadings^a^			Cronbach α^b^	AVE^c,d^	CR^e,f^
**PE^g^**					
	**PE** **indicator**				
		0.951	.957	0.918	0.971
		0.962	.957	0.918	0.971
		0.961	.957	0.918	0.971
**EE^h^**					
	**EE indicator**				
		0.913	.797	0.698	0.872
		0.662	.797	0.698	0.872
		0.906	.797	0.698	0.872
**FC^i^**					
	**FC indicator**				
		0.953	.921	0.853	0.946
		0.950	.921	0.853	0.946
		0.866	.921	0.853	0.946
**SI^j^**					
	**SI indicator**				
		0.261	.460	0.455	0.978
		0.983	.460	0.455	0.978
		0.912	.460	0.455	0.978
**BI^k^**					
	**BI indicator**				
		0.915	.918	0.860	0.948
		0.972	.918	0.860	0.948
		0.893	.918	0.860	0.948

^a^Outer loadings ≥0.5 indicate indicator reliability. With a reflective model, internal consistency is measured by Cronbach α.

^b^Cronbach α ≥.7 indicates good indicator reliability.

^c^AVE: average variance extracted.

^d^AVE ≥0.5 indicates convergent validity.

^e^CR: composite reliability.

^f^CR ≥0.5 indicates good internal consistency.

^g^PE: performance expectancy.

^h^EE: effort expectancy.

^i^FC: facilitating conditions.

^j^SI: social influence.

^k^BI: behavioral intentions.

**Table 5:** Intercorrelations between study variables measured by the FLC^a^ and HTMT^b.c^.

Measures and latent variables			BI^d^		EE^e^		FC^f^		PE^g^		SI^h^
**FLC**											
	BI	0.927		—^i^		—		—		—	
	EE	0.467		0.835		—		—		—	
	FC	0.378		0.529		0.924		—		—	
	PE	0.220		−0.262		0.062		0.958		—	
	SI	0.469		0.305		0.403		0.166		0.675	
**HTMT**											
	BI	—		—		—		—		—	
	EE	0.486		—		—		—		—	
	FC	0.371		0.695		—		—		—	
	PE	0.224		0.316		0.122		—		—	
	SI	0.574		0.595		0.468		0.391		—	

^a^FLC: Fornell-Larcker Criterion.

^b^HTMT: heterotrait-monotrait ratio.

^c^Diagonals are the square root of the average variance extracted of the latent variables and indicate the highest in any column or row.

^d^BI: behavioral intentions.

^e^EE: effort expectancy.

^f^FC: facilitating conditions.

^g^PE: performance expectancy.

^h^SI: social influence.

^i^Not applicable.

**Table 6:** Structural model evaluation and hypothesis testing (prediction of BI^a^).

Relationship	Standard β (SE)	T value	*P* value	Effect size (*f*^2b^; 95% CI)
PE^c^>BI	.293 (0.544)	0.869	.39	0.112 (−0.808 to 1.249)
EE^d^>BI	.455 (0.444)	0.812	.40	0.215 (−0.819 to 0.747)
SI^e^>BI	.278 (0.337)	0.734	.47	0.104 (−0.446 to 0.799)
FC^f^>BI	.007 (0.364)	0.014	.99	0.004 (−0.569 to 0.887)

^a^BI: behavioral intentions.

^b^Effect size (*f*^2^) values <0.02 denote small effect size or predictive relevance.

^c^PE: performance expectancy.

^d^EE: effort expectancy.

^e^SI: social influence.

^f^FC: facilitating conditions.

**Table 7:** Results of the Wilcoxon signed-ranks test for pre-post changes in latent variable ranks^a^.

Latent variables	*Z* score (SE)	Significance (*P* value)
BI^b^	33 (9.715)	.57
EE^c^	65 (11.214)	.004^d^
FC^e^	32 (15.843)	.20
PE^f^	39.5 (11.147)	.56
SI^g^	13.5 (8.178)	.27

^a^Total: *Z* score 52.5 (SE 14.283); *P*=.62 (significance).

^b^BI: behavioral intentions.

^c^EE: effort expectancy.

^d^Statistical significance at *P*=.05.

^e^FC: facilitating conditions.

^f^PE: performance expectancy.

^g^SI: social influence.

### Thematic Analysis

#### Overview

Thematic analysis was conducted by analyzing the responses to the open-ended questions from the UTAUT questionnaires and interviews after 3MDR for the participants, therapists, and operators. Three themes emerged: (1) feasibility and function, (2) technical support, and (3) tailored immersion (Table 8).

**Table 8:** Thematic analysis results of open-ended questions from the Unified Theory of Acceptance and Use of Technology questionnaire and qualitative interviews.

Theme	Illustrative quote
Feasibility and function	“The look of the program, it just looks a bit outdated and its small, can be more attractive to make it more user-friendly...but it does work, then with the new session [we] have a.PDF file with all the pictures and associations and units of distress, walking speed average...[with] this new system [documentation] looks way better—not just a sheet with all the information...being able to download the data in a clean way.” [T13] “Improve resolution of photos.” [T7]
Technical support	“I suppose that there is a lot of moving pieces, so it is technology dependant, so if something goes down and there is a glitch it throws a monkey wrench in it. You need ‘techy’ people.” [T1] “I would just give the pictures to the operator, everything worked fine. I feel, like I said, I feel comfortable being in that, or working with that technology.” [T5]
Tailored immersion	“I want to make it more personalized, now the virtual reality has been chosen by a developer who thinks this is the correct virtual environment, but I think this is the wrong way around. I think we should let our patients decide which virtual environment they want to walk in.” [T20] “When people walk fast they are also walking fast to the picture. I would like to have an option to increase the length of the tunnel, so people can walk fast, but so the photo doesn’t come up as fast.” [T21]

#### Feasibility and Function

Overall, the CAF military members, veterans, therapists, and operators found that 3MDR was feasible and functional within their given environments for the purpose of the research study. That said, the end users, particularly therapists and operators, noted a number of items that they felt could be improved to enhance the overall functioning and patient experience in hopes that it would lead to better outcomes. Improvements to the technology that would assist with the delivery and functionality of 3MDR for therapists and operators included more streamlined documentation, ease of downloading of data, and overall intuitiveness of the software. This theme fits within the construct of EE, as many of the suggested modifications and improvements targeted to improve the ease of use of the overall 3MDR system elements [19]. In addition, some aspects, such as improving the quality of the images, correlate with PE, for which improved performance or outcomes is a potential goal.

#### Technical Support

Similarly, CAF military members, veterans, therapists, and operators identified that they felt satisfied with the level of technical support they received. The participants felt that the team was knowledgeable and able to operate the hardware and software with a high level of competence. When there were glitches, they could be troubleshooted and resolved within a reasonable amount of time. Therapists generally felt that the 3MDR operator was their main source of technological support and that the operators were proficient in providing this. The operators generally felt that they were able to receive support from other VRE operators globally, who had also used 3MDR. The experience of the operators with software and hardware support from vendors was variable, with some reporting that the vendor’s expertise with technology geared toward physical health interventions rather than mental health was a barrier. The theme of technical support falls under the construct of FC as it regards that organizational and technical infrastructure exists to support the use of the system [19].

#### Tailored Immersion

The desire to customize the experience of 3MDR for the patient through technology was the strongest theme among the CAF military members, veterans, therapists, and operators. The vast majority of feedback regarding the technological aspects of 3MDR and the associated VRE provided recommendations on how population-specific stakeholders should be used to adapt the hardware and software for future tailoring of the 3MDR intervention. 3MDR therapists desired to have the ability to tailor aspects of the software, such as the length of the tunnel, length of the image exposure, default VRE, and the number of images, in real time based on their clinical observations and needs. Therapists and participants also identified the need to make 3MDR software and hardware accessible to those who may have reduced mobility and who may use a wheelchair. Tailored immersion is correlated with the construct of PE. The desired customization of software and hardware stems from the belief that the system will help the patient or participant attain gains in performance or improved outcomes regarding their PTSD symptoms.

## Discussion

### Principal Findings

In this preliminary study, the UTAUT model was used as the theoretical foundation for understanding the behavioral intention of CAF military members and veterans with crPTSD, as well as their therapists and operators, in using 3MDR. On the basis of the study results, 3MDR delivered within a VRE appears to be promising as a feasible, usable, and accepted technological intervention for end users, with EE being the most notable predictor of BI and deemed to be the most important to end users. Although the qualitative data support this, it is worth noting that none of the latent variables yielded statistical significance with PLS-SEM. There was also no significant difference detected between the patient end user (CAF military members and veterans) and the health care end user (therapists and operators) scores for PE, EE, SI, FC, or BI. The analysis of the open-ended questions and qualitative interviews revealed several subthemes that can be attributed to the latent variables, including EE, PE, and FC of the UTAUT, as well as BI as a construct. To triangulate the quantitative and qualitative data, possible explanations for the results were formulated [37].

Overall, end users rated all the latent variables (PE, EE, FC, and SI) and BI favorably for the technological aspect of 3MDR. The data demonstrated that participants generally agreed or strongly agreed with the statements made in the UTAUT questionnaires, especially for the variables of PE, EE, and FC. The results of PLS-SEM analysis demonstrated good internal consistency, convergent validity, composite reliability, and discriminant validity of the indicators, with a moderate predictive accuracy of the model.

EE is the degree of ease associated with the use of a system [19]. EE had the largest path coefficient and effect size, indicating that it was the strongest predictor of BI when compared with the other latent variables. A statistically significant increase in EE was noted in the pre-post analysis, whereas the pre-post changes in the other latent variables were nonsignificant. A change of <5% (0.82%) in the before and after scores indicates that the expectations of the technological aspects of 3MDR were generally met or exceeded. This was further verified by a statistically nonsignificant difference in pre- and post-UTAUT questionnaire results based on the Wilcoxon signed-ranks test.

This is contrary to the hypothesis that FC is the strongest predictor based on previous literature regarding patients and health care professionals in a North American context [12,21]. However, previous literature has hypothesized that military organizations’ approach to technology is to measure and maximize operator performance to increase system efficiency, which translates to success in military missions [23]. This may provide some insight into why the CAF military members and veterans felt that ease of use or efficiency was the most important aspect of a favorable user experience. Many of the qualitative quotes within the subthemes fell into the category of EE, demonstrating that 3MDR within the VRE was perceived as easy to use by all study end users, which was of utmost importance. Given that both the qualitative and quantitative data demonstrated that this latent variable was important to the end users in the study, it should be considered further in modifications and adaptations to 3MDR and the used VRE.

As previously mentioned, PE refers to the degree to which an individual believes that using the system will help the person attain gains in performance [19]. In the context of 3MDR within a VRE, performance is measured and communicated via clinical outcome measures, live biofeedback data, feedback from the therapist or patient, and the subjective experience of PTSD symptoms after the intervention session [9]. During the 3MDR platform sessions, the participant is limited to their intrinsic subjective insight to speculate on their performance without any immediate feedback on their performance. Only after the actual 3MDR sessions would the military member or veteran notice any changes in their PTSD symptoms and attribute them to 3MDR and thus their PE. In addition, therapists and operators do not have any direct feedback during the sessions on their own performance unless there is a technological event during the session in which they cannot reconcile, such as a technological malfunction. These notions may be logical explanations as to why PE did not register as an important factor in BI and did not demonstrate a significant pre-post change. It should also not be ignored that the indicator variables for PE demonstrated issues with lateral collinearity, as demonstrated by their variance inflation factors, which may affect the accuracy of this latent variable.

SI is the degree to which an individual perceives that it is important that others believe that they should use the new system [19]. As 3MDR was administered within a research study with limited persons present and confidentiality was maintained, it is unlikely that the patients perceived SI as being relevant specifically to the 3MDR technology. This was demonstrated to be an accurate hypothesis as SI was the least influential latent variable in the prediction of BI. Previous studies have demonstrated that SI is less likely to factor into the perceived acceptance and usability of health care technology than other latent variables for health care professionals [14,21,22]. On the basis of the previous literature, health care providers have demonstrated emphasis on PE, EE, and FC as constructs that influence BI and, therefore, use.

FC is the degree to which an individual believes that organizational and technical infrastructure exists to support the use of the system [19]. This latent variable did not have much impact on BI based on PLS-SEM, as predicted in the hypothesis. In the qualitative themes, technical support came through as a priority for end users in different ways, which would fall under the FC. For the 3MDR therapists, having the belief that the 3MDR operator had the infrastructure and knowledge to effectively run the VRE and facilitate the 3MDR software was important. Subsequently, this confidence in the operator was also transmitted to the participant, who had confidence in both the operator and the therapist that any technological challenges could be quickly and seamlessly fixed. It is logical that the 3MDR participants felt supported by their therapists, operators, study team, organization, and other facilitators in the immediate environment.

### Limitations of Study

Although PLS-SEM is ideal for exploratory research and flexible with its nonparametric lack of assumptions regarding data distribution, several limitations need to be considered. First, measurement errors always exist to some degree and are challenging to quantify accurately. PLS-SEM bias refers to the tendency of the path model relationships to be frequently underestimated, whereas the parameters of the measurement model, such as the outer loadings, are overestimated when compared with covariance-based SEM. Measurement errors can also be introduced by variables such as the participants’ understanding of the questionnaire items. In addition, the administrative burden of the study, when combined with other outcome measures attributed to the greater clinical trial with which this study was affiliated, may have caused some participants to rush through final questionnaires or experience fatigue and a reduced level of engagement. Second, the lack of global goodness-of-fit measures is an unavoidable drawback of PLS-SEM. Finally, the small sample size because of COVID-19 related shutdowns made it impossible to incorporate the moderator variables of age and gender, as was originally planned in the research model, and the desired sample power was not met (Figure 1). Despite these shortcomings, it is important to discuss preliminary findings and address technology acceptance and usability early in the process of implementing novel interventions to ideally detect and avoid problems with end user buy-ins, which could hinder the uptake and spread of potentially valuable innovations in health care settings. The research team will be continuing data collection to reach the desired sample size in the future.

### Future Research

The technology acceptance and usability of 3MDR within a VRE, as well as other interventions using technology, warrant evaluation within military and civilian health care contexts and at multiple user levels, including the patient, health care professional, and organization. This also extends to the use of web-based health care technologies where the patient is in a separate location from the health care professionals—a practice that is becoming increasingly widespread, especially in the wake of a global pandemic [17,18]. Future research that could support the advancement of 3MDR might include studies with larger samples to allow for the ability to incorporate moderator variables such as age, gender, voluntariness of use, and experience, as well as to use other models of technological acceptance and usability. In addition, the exploration of the utility of the UTAUT as a model for health care technology warrants continued investigation in both civilian and military settings, where research is extremely scarce [22,23]. Equally, the improvement of 3MDR hardware and software has evolved at a rapid pace, further complicating accurate research. Since this study, a number of the themes mentioned by end users have already been addressed, and tailored immersions and customizability of the hardware and software for other trauma-affected populations are being trialed. As 3MDR evolves to be more accessible and uses new hardware such as wearable VR, acceptability and usability perceptions of end users will need to be considered. In addition, a cost analysis of 3MDR would also be beneficial to study, as this also affects the implementation of technological innovation in health care. Finally, future research is needed to address its acceptability and effectiveness among other trauma-affected populations, which may improve accessibility to 3MDR.

### Conclusions

Numerous military personnel and veterans from around the globe who have returned from deployment continue to struggle with the symptoms of PTSD. Despite the plethora of research, publications, and attention that PTSD has received in recent years, many questions remain regarding the complexities of treating the psychological symptoms attributed to this diagnosis. 3MDR challenges traditional conventions and configurations. It is important to incorporate the study of technology acceptance and usability into the implementation of novel VR-supported health care processes to ensure that technological advances aimed at assisting patients will be embraced by the primary intended users. This is important at the micro, meso, and macro levels, especially within unique organizational contexts such as military and health care systems. 3MDR appears to be a promising intervention for crPTSD, with good acceptability by end users, including CAF military members and veterans, as well as 3MDR therapists and operators. The future for the usability of 3MDR is promising, and new and exciting intervention avenues for crPTSD will emerge because of continued research. As civilian and military health care systems increasingly integrate technological innovations to improve the services and care provided to their patients, research must continue to address questions of technological acceptance of the intervention before its wide-scale adoption.

## Abbreviations

3MDR: multimodal motion-assisted memory desensitization and reconsolidation
AVE: average variance extracted
BI: behavioral intentions
CAF: Canadian Armed Forces
CAREN: Computer Assisted Rehabilitation Environment
crPTSD: combat-related posttraumatic stress disorder
EE: effort expectancy
FC: facilitating conditions
PE: performance expectancy
PLS: partial least square
PLS-SEM: partial least squares structural equation modeling
PTSD: posttraumatic stress disorder
REDCap: Research Electronic Data Capture
SEM: structural equation modeling
SI: social influence
T0: time point 0
T1: time point 1
UTAUT: Unified Theory of Acceptance and Use of Technology
VR: virtual reality
VRE: virtual reality environment
